# A Holistic Approach to Circular Bioeconomy Through the Sustainable Utilization of Microalgal Biomass for Biofuel and Other Value-Added Products

**DOI:** 10.1007/s00248-024-02376-1

**Published:** 2024-04-25

**Authors:** Ganesan Ezhumalai, Muthukrishnan Arun, Arulmani Manavalan, Renganathan Rajkumar, Klaus Heese

**Affiliations:** 1 Department of Environmental Sciences, School of Life Sciences, Bharathiar University, Coimbatore, Tamil Nadu 641046 India; 2 Department of Biotechnology, School of Life Sciences, Bharathiar University, Coimbatore, Tamil Nadu 641046 India; 3 Department of Cariology, Saveetha Dental College and Hospitals, Saveetha Institute of Medical and Technical Sciences, Chennai, Tamil Nadu 600077 India; 4 Graduate School of Biomedical Science and Engineering, Hanyang University, 222 Wangsimni-ro, Seongdong-gu, Seoul, 133791 Republic of Korea

**Keywords:** Biodiesel, Biofertilizer, Biofuels, Circular bioeconomy, Feed supplement, Lipid

## Abstract

**Abstract:**

Emissions from transportation and industry primarily cause global warming, leading to floods, glacier melt, and rising seas. Widespread greenhouse gas emissions and resulting global warming pose significant risks to the environment, economy, and society. The need for alternative fuels drives the development of third-generation feedstocks: microalgae, seaweed, and cyanobacteria. These microalgae offer traits like rapid growth, high lipid content, non-competition with human food, and growth on non-arable land using brackish or waste water, making them promising for biofuel. These unique phototrophic organisms use sunlight, water, and carbon dioxide (CO_2_) to produce biofuels, biochemicals, and more. This review delves into the realm of microalgal biofuels, exploring contemporary methodologies employed for lipid extraction, significant value-added products, and the challenges inherent in their commercial-scale production. While the cost of microalgae bioproducts remains high, utilizing wastewater nutrients for cultivation could substantially cut production costs. Furthermore, this review summarizes the significance of biocircular economy approaches, which encompass the utilization of microalgal biomass as a feed supplement and biofertilizer, and biosorption of heavy metals and dyes. Besides, the discussion extends to the in-depth analysis and future prospects on the commercial potential of biofuel within the context of sustainable development. An economically efficient microalgae biorefinery should prioritize affordable nutrient inputs, efficient harvesting techniques, and the generation of valuable by-products.

**Graphical Abstract:**

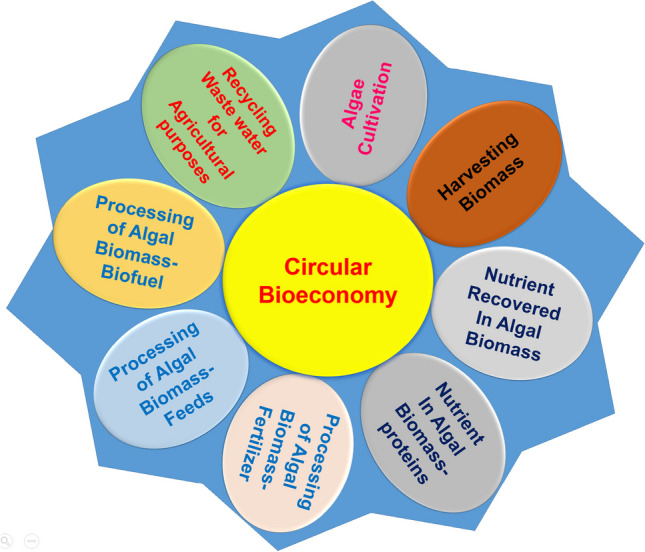

## Introduction

Global warming, flooding, glacier melting, and rising sea levels are outcomes of greenhouse gas emissions from industrial and transportation activities. The use of fossil fuels for energy, driven by the industrial revolution, has led to climate change and energy insecurity [1]. However, fossil fuels are non-renewable due to their limited supply and rapid depletion from excessive consumption. This reliance on fossil fuels worsens climate change and drives the search for alternative energy sources [2]. Population growth and improved living standards have increased the consumption of natural resources like wood, oil, and water, leading to water and air pollution from various sectors [3].

The urgent need for clean and sustainable energy sources to address environmental issues has led to the exploration of thermal, tidal, hydro, solar, mechanical, and nuclear energy sources [2]. Eco-friendly technologies have reduced India’s reliance on fossil fuels for power generation from 68 to 63.05% [4]. However, this shift has increased global greenhouse gas emissions, primarily CO_2_, which are projected to rise by 1.3% per year between 2005 and 2011 [1, 5]. Microalgae have significant potential as biological resources in industries like medicine, healthcare, feed, and fuel. With over 200,000 species, they are the largest primary producers on Earth [4].

Aquatic algae, found in freshwater, brackish, spring, and salt lake habitats, have potential as a valuable feedstock for bioproducts due to their ability to capture carbon and remove nitrogen (N) and phosphorus (P) from wastewater [6, 7]. Microalgae are versatile, used for energy, biofuels, nutrition, health, cosmetics, and wastewater treatment, fixing nitrogen, carbon, and heavy metals [8]. For instance, current research has focused on integrating wastewater treatment with suitable algae cultivation species, like *Chlorella pyrenoidosa* and *Chlamydomonas polypyrenoideum* for effective bio-oil production [1, 9].

Microalgae have been studied extensively as a viable alternative feedstock for biofuels [10], with higher growth rates and yields than conventional plants [11]. They offer rapid growth, high photosynthetic efficiency, high biomass production, and a high lipid content, requiring less land area and not competing with food sources [12]. Microalgal oil can be converted into biodiesel, with higher productivity per acre of land than conventional feedstocks, utilizing marginal water sources like wastewater and agricultural runoff [13]. Effective cultivation methods are crucial for cost-effective operations, using photobioreactors (PBR) and raceway ponds.

Microalgae can grow in marginal terrain using water sources unsuitable for agriculture, with a potential oil yield 5–20 times higher than palm oil on an equal area basis [14]. Algal biomass contains lipids, proteins, and carbohydrates, varying by species and cultivation [13]. Microalgae can produce direct combustion crude algal oil for transportation fuels and biodiesel through solvent extraction and trans-esterification [15].

Anaerobic processes like biohydrogen production, biogas digestion, and bioethanol generation can utilize microalgae [2]. The remaining biomass from oil extraction can be used for biomethane, bioethanol, biosorbents for wastewater treatment, and biofertilizer [11, 16–18]. Microalgae can contribute to a circular bioeconomy, producing biofuels, biohydrogen, and value-added products [3]. Lipid-extracted algal biomass can be used for hydrogen and methane generation, fermentable sugar production, bioethanol development, biofertilizer creation, and animal feed production [19].

Genetic modification presents viable solutions to challenges encountered in the production of bioenergy derived from algae. The realization of large-scale production of genetically modified (GM) algal biomass, referred to as fourth generation biofuel (FGB), is confronted by several hurdles including ecological risks, high production costs, concerns about legitimacy, and suboptimal growth rates, among other factors [20, 21]. Microalgae-derived biofuels hold great promise for fostering a sustainable bioeconomy, with the pivotal determinants of cost residing in oil content and biomass yield during FGB production. Novel insights delve into evolving trends in genetic modification aimed at augmenting both oil accumulation and biomass yield, thereby propelling advancements in the bioeconomy. The substantial enhancement of these factors significantly amplifies the economic success and net present value of FGB production. The utilization of GM strains in fully lined open raceway ponds may incur costs up to 25% higher than their unlined counterparts. The implementation of a plastic hoop air-supported greenhouse for pond cultivation is estimated at $60,000 per hectare. The competitiveness and profitability of large-scale GM biomass cultivation hinge on techno-economic and socioeconomic drivers, necessitating further research and prolonged studies to comprehend pivotal influencing mechanisms. The burgeoning applications of genetic engineering in biofuel development, especially in FGB derived from GM algae biomass, captivate attention. However, persistent challenges demand a nuanced exploration of technical aspects in genetic modification operations, particularly those concerning GM algal biomass. Issues such as diffusion risk, regulatory frameworks, biosafety, and the intricacies of global consensus efforts at international levels underscore the need for comprehensive scrutiny [3, 22–24].

The application of molecular and genetic tools emerges as a potent strategy to enhance the yield of biomolecules in microalgae. Recent advancements in metabolic engineering focus on the overexpression or knockout of specific genes governing enzymes within biosynthetic pathways of interest, with particular emphasis on fatty acid pathways in numerous studies. Despite this progress, the development of new genetic toolkits tailored for diverse microalgae species is imperative for their effective integration into industrial platforms. Additionally, the synergy of computational biology, bioinformatics, and multiomics datasets is poised to play a pivotal role in enhancing the biorefinery products of microalgae [25].

The challenges facing the widespread adoption of microalgae biofuels are manifold. High production costs impede their commercialization on a large scale, while limitations in scalability and technological constraints in lipid extraction methods hinder efficient production processes. Access to suitable cultivation sites, water resources, and nutrients poses significant challenges regarding resource availability [26]. Ensuring economic viability entails developing cost-effective cultivation and processing methods, as well as strategies for generating revenue from by-products [27]. Regulatory and policy barriers must be navigated to meet compliance with environmental standards and land use policies [28]. Addressing environmental concerns, such as impacts on water quality and biodiversity, is crucial for sustainable production practices [29]. Achieving market acceptance and consumer adoption requires overcoming perceptions of performance, safety, and compatibility with existing infrastructure [30]. Research and development efforts must focus on addressing gaps in current knowledge and innovation to overcome existing challenges [31]. Integrating microalgae biofuel production systems into existing energy infrastructure and supply chains presents logistical and technical challenges. Long-term sustainability considerations, including land use competition and lifecycle environmental impacts, necessitate strategic planning and mitigation measures [32]. The novelty of this review article lies in its comprehensive examination of the challenges and opportunities surrounding microalgae biofuels production. While existing papers may have touched upon certain aspects of microalgae biofuels, this review uniquely synthesizes current research on lipid extraction methods, value-added product development, and commercial-scale production challenges [29, 33]. Moreover, it delves into the integration of microalgae biofuels within the broader context of sustainable development, emphasizing the importance of economic efficiency, resource optimization, and environmental stewardship. By offering a holistic perspective on the subject, this review article provides valuable insights for researchers, policymakers, and industry stakeholders seeking to advance the field of microalgae biofuels in a sustainable and economically viable manner.

## Lipid Extraction Methods

Extracting lipids from microalgae can be easy in some cases, but in others, the lipids may be enclosed within cells and protected by strong cell walls, making extraction harder. While microalgal lipid extraction has low costs, it also has downsides like poor mass transfer, limited yield, and long extraction times [34]. The cost-effectiveness and efficiency of lipid extraction are crucial for microalgal biodiesel production, as different strains of microalgae have unique structures and compositions. Therefore, a more efficient and affordable method of lipid extraction is needed for large-scale biodiesel production. Furthermore, the complex and hard cell walls of microalgae can be broken down by a variety of mechanical, chemical, and enzymatic treatments, which makes it easier for solvents to penetrate and extract the lipids [5, 35].

Several methods for lipid extraction are available, such as the Bligh and Dyer method using a 2:1 ratio of methanol to chloroform [36], the Soxhlet method with hexane [37], ultrasound-assisted extraction [38], and microwave extraction. After each process, the microalgae biomass extract is filtered to remove any remaining biomass. The leftover crude lipid is then measured gravimetrically after the solution has evaporated. The Soxhlet extraction method is generally considered the most effective, achieving 100% recovery of all lipids in microalgae. Some studies suggest that using a solvent mixture of methanol and chloroform yields more lipid extract compared to hexane extraction. Unlike hexane, which selectively dissolves non-polar lipids, the methanol-chloroform mixture can extract both neutral and polar lipids. A more promising approach includes combined enzymatic and mechanical/solvent-free extraction methods to lower solvent usage and energy consumption for extracting lipids efficiently [5, 39].

To produce sustainable fuel with smooth engine performance, microalgae oil’s high viscosity needs to be reduced during biodiesel production to prevent engine damage from oil sludge accumulation [34]. Transesterification is commonly used to lower the viscosity of microalgae oil by turning it into fatty acid methyl esters (FAME) or biodiesel. The efficiency of the transesterification and purification procedures are mainly based on the number of the biodiesel’s measurable characteristics, which includes its water content, flash point, methanol concentration, and glyceride content. The remaining factors, such as iodine value, viscosity, cetane number, cold properties, distillation temperature, and polyunsaturated fatty acid, are primarily determined by the fatty acid composition [5, 40]. Inexpensive alcohols like methanol and ethanol are suitable for synthesizing biodiesel due to their affordability and favorable properties [41]. Typically, methanol or ethanol is used as the alcohol source in the transesterification reaction [42]. A catalyst is needed to facilitate the reaction between the parent oil (triglyceride) and the short-chain alcohol to produce FAME and glycerol. Catalysts like acids (hydrochloric, sulfuric, phosphoric, sulfonic), bases (sodium hydroxide, potassium hydroxide), or enzymes (lipase) can enhance the reaction rate and product yield. Base catalysts can achieve high FAME yields quickly, at low pressure and temperature [34, 42–45]. Heterogeneous catalysts like magnesium oxide, barium oxide, calcium oxide, and strontium oxide have successfully catalyzed transesterification reactions, being non-corrosive, environmentally friendly, and cost-effective. Heterogeneous acid catalysts are also studied, unaffected by water and free fatty acids during transesterification. However, extreme reaction conditions like high alcohol-to-oil ratio and temperature are necessary for these catalysts to accelerate the reaction [5].

## Sustainable Utilization of Microalgal Biomass

**Fig. 1:**
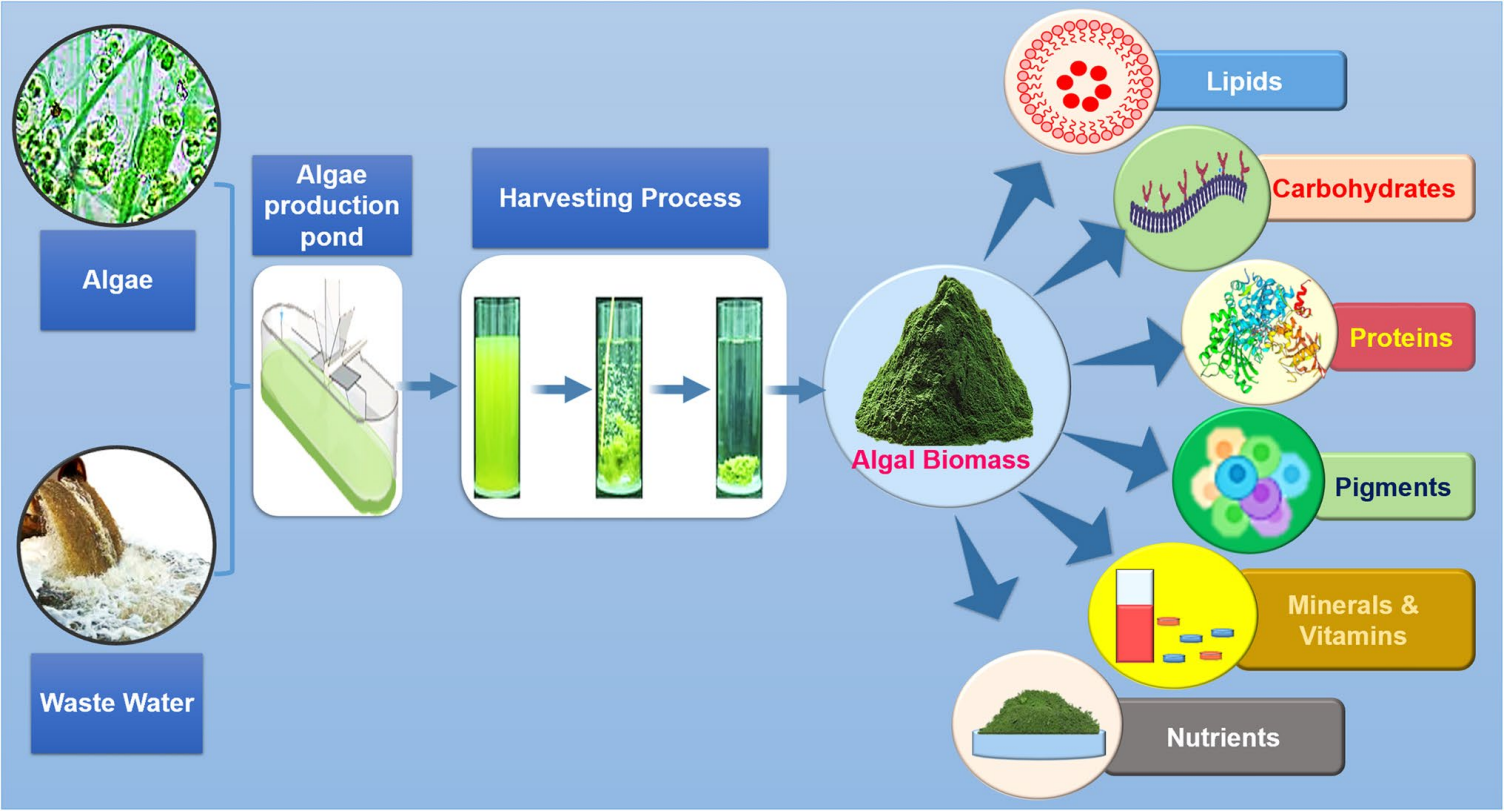
Schematic representation of the routes to valorise microalgae biomass from cultivation to products. Microalgae are cultivated using wastewater in the bioreactor, and biomass is extracted. Using the microalgal biomass, biofuel and other value-added products were produced

Microalgae are a rich source of proteins, carbohydrates, lipids, vitamins, and antioxidants, making them valuable for producing high-value products [46, 47]. The microalgae biomass obtained from harvesting can be used for biofuels, animal feed, biofertilizers, and bioactive products (Fig. 1). However, the development cost of microalgae bioproducts remains high [48]. To address this, it is important to explore using wastewater nutrients for microalgae cultivation, which can reduce production costs and advance the industry [49]. A research investigation has unveiled that vegetable waste can serve as a valuable nutrient source for cultivating microalgae, thereby mitigating the production costs associated with biofuel synthesis [50]. Various case studies have elucidated diverse methodologies in utilizing vegetable waste as a pivotal nutrient source for microalgae cultivation, thereby mitigating the production costs entailed in biofuel synthesis. Particularly noteworthy, Chaudhary et al. (2017) showcased remarkable biomass productivity rates spanning from 3.10 to 4 g m^−2^ d^−1^ while cultivating a mixed microalgae culture across various wastewater variants, encompassing livestock wastewater, greywater, and anaerobically digested slurry [51]. The proposition of microalgae cultivation utilizing nutrient-rich wastewater emerges as a strategic avenue for cost reduction, underscored by its potential efficacy. Nonetheless, notwithstanding these auspicious discoveries, the constrained concentration of microalgae within growth media derived from wastewater, as underscored by Rana et al. [52], poses a substantial impediment to the commercial feasibility of microalgal biofuel production. The rectification of this predicament assumes paramount importance in unlocking the complete potential of microalgae cultivation in wastewater for the purpose of sustainable biofuel production.

It is crucial to not only focus on algal-based biofuel technologies, as de-oiled microalgal biomass (DMB) has potential for creating value-added bioproducts and environmental benefits [1]. Converting biomass into biofuel offers an opportunity to produce additional chemicals and biomaterials from residual biomass, increasing its value and reducing waste. A range of primary and by-products derived from algae are commercially viable, supporting a green economy (Table 1). Using pure and raw algal biomass can produce high-value products like biodiesel, bioethanol, biohydrogen, and biogas, as well as cosmetics, medicines, feed, and fertilizers [1].

**Table 1:** Biochemical content of various microalgae species

Serial No	Name of the microalgal strain	Name of the biomolecules	Biochemical content (%)	Reference
1	*Chlorella vulgaris*	Lipid Protein Pigment	42 26 0.74	[53]
2	*Schizochytrium mangrovei*	Lipid Protein Carbohydrates	30 10 30-40	[54]
3	*Chlorella pyrenoidosa*	Protein Carbohydrates	10 30-40	[54]
4	*Dunaliella salina*	Lipid Protein Carbohydrates	11 55 30	[55]
5	*Nannochloropsis salina*	Lipid Protein Carbohydrates	28 35 28	[55]
6	*Scenedesmus* sp.	Lipid Protein Carbohydrates	23.62 - 42.68	[56]
7	*Scenedesmus obliquus*	Lipid Protein Carbohydrates	30.85 19.52 35.05	[57]
8	*Ankistrodesmus falcatus*	Lipid Protein Carbohydrate	35.9 30.59 33.88	[57]
9	*Chlorella sorokiniana*	Lipid Carbohydrate Protein	19.8 20.0 25.5	[58]

Lipid extraction from algal biomass is mainly used for biodiesel, but bioplastics can also be derived from lipids, proteins, and carbohydrates. Aqueous hydrolysate residues and cell debris from biofuel conversion can be used to make proteins, glucose, peptides, and amino acids. DMB can then be used to make proteins for food and feed, bioplastics, foams, glue, and biocomposites. Stoichiometric hydrogen production has been reported from residual algal biomass [59], and a range of lipid content has been observed in *Chlamydomonas reinhardtii* strain D1 [60]. Table 2 provides a comprehensive overview of the utilization of lipid-extracted algal biomass [66]. Moreover, the residual biomass resulting from the transesterification process serves as a by-product and can be repurposed for animal feed, containing glycerol, carbohydrates, and proteins [67]. It is noteworthy that methane and hydrogen production have been documented from lipid-extracted algal biomass [66, 68], and lipids have been successfully produced from both lipid-extracted algal biomass and molasses [69]. Furthermore, biogas generation has been achieved through the anaerobic co-digestion of de-oiled microalgae [70].

**Table 2:** Utilization of lipid-extracted algal biomass for variety of applications

Serial No	Culture	Cell disruption methods	Biomass	Technical details	Value-added product	Ref.
1	*Acutodesmus obliquus*	Chemical method	8.9 g L^−1^	Transesterification of lipid	Biodiesel (98.75%)	[61]
2	*Schizochytrium mangrovei*	Soxlet	Not mentioned	Transesterification of lipid	Biodiesel (68.98 %)	[62]
				Utilizing of by-product (crude glycerol) during transesterification process	Glycerol as a carbon source for algae cultivation	
3	*Chaetoceros* sp*.*	Chemical method	0.217 g L^−1^	Transesterification of lipid	Biodiesel (38.49%)	[63]
				Utilizing lipid extracted biomass used for measuring antioxidant assay	Antioxidative and antibacterial activity	
4	*Skeletonema* sp*.*	Chemical method	0.184 g L^−1^	Transesterification of lipid	Biodiesel (64.45%)	[63]
				Utilizing lipid extracted biomass used for measuring antioxidant assay	Antioxidative and antibacterial activity	
5	*Thalassiosira* sp*.*	Chemical method	0.166 g L^−1^	Transesterification of lipid	Biodiesel (52.73%)	[63]
				Utilizing lipid extracted biomass used for measuring antioxidant assay	Antioxidative and antibacterial activity	
7	*Scenedesmus bijugatus*	Soxhlet extraction	0.26 g L^−1^	Esterification and transesterification process	Biodiesel (0.21 g/g)	[64]
				Utilizing lipid extracted biomass used for fermentation process	Bioethanol (0.15g/g)	
8	*Auxenochlorella protothecoides*	Anaerobic digestion	Not mentioned	Digestion process (biodigesters)	Biogas (55%) Methane (53%)	[64]
9	*Chlorella pyrenoidosa*	Pyrolysis	Not mentioned	Thermal decomposition process	Biochar (51.23 %) Biogas (13.44 %) Bio oil (35.33 %)	[65]

### Biomethane

Biomethane, a renewable natural gas generated from anaerobic digestion of organic materials like agricultural waste, sewage, and food waste, is considered an eco-friendly alternative to traditional fossil fuels. De-lipidified microalgae residue with a high C/N ratio aids in producing methane-rich biogas. Research has explored biomethane production from microalgal biomass, including lipid-extracted biomass [71], and optimizing parameters for methane synthesis from various algal strains. These experiments resulted in improved biomethane generation, with yields ranging from 140 to 380 ml of CH_4_/g—volatile solids. Biomethane, with its high calorific value, enhances overall biorefinery efficiency, serving purposes like cooking, heating, and lighting. A study revealed higher energy efficiency and lower greenhouse gas emissions in biomethane production from food waste and sewage sludge, making it an effective waste management strategy [72]. Others highlighted the economic and environmental sustainability of biomethane production using agricultural waste, reducing competition for resources [73]. Challenges in biomethane production include ammonia production from high N content, which can be mitigated through co-digestion and pre-treatment methods like enzyme-pre-treated DMB [71]. Thus, biomethane holds promise as a renewable energy source, with potential benefits from utilizing food waste and sewage sludge as feedstocks, but addressing environmental impacts is crucial for its sustainable production and utilization [74].

### Biohydrogen

Biohydrogen is a renewable energy produced through the biological conversion of organic matter like agricultural waste, sewage, and food waste. It is a sustainable and eco-friendly alternative to fossil fuels, especially in transportation. A recent study assessed biohydrogen production potential from different sources. They found that food waste and sewage sludge had higher energy efficiency and lower greenhouse gas emissions than grass and agricultural waste [75]. This could aid waste management and process sustainability. Algae have great potential for biohydrogen production due to their fast growth, high biomass, and adaptability. They also reduce greenhouse gases and water usage compared to traditional methods. However, challenges exist in biohydrogen production. A study on cost and scalability showed that it is still costly and limited by feedstock availability and conversion efficiency. More research is needed to make biohydrogen production cost-effective and scalable.

After recovering lipids, leftover biomass has carbohydrate and proteins that hydrogen-producing microbes need. Extracting oil from dried microalgae biomass is challenging for conversion into hydrogen or other fuels. Studies show that pre-treating dried microalgae biomass is crucial for releasing cellular carbon, enabling microbes to produce hydrogen or methane. Different pre-treatment methods like chemical, mechanical, and thermochemical techniques improve hydrogen production. For example, thermo-alkaline pre-treatment at 100 °C with 8 g/L NaOH increases hydrogen yield threefold, to 45.54 mL/g of inflammable solid [76]. It also enhanced carbohydrate and protein solubilization. Biohydrogen production from dried mixed microalgae biomass using acidogenic bacteria can achieve specific hydrogen yields of 4.9, 3.3, 3.0, and 2.4 mol/kg chemical oxygen demand from different untreated and acid-treated biomass [66].

### Bioethanol

Bioethanol, a renewable biofuel produced from plant-based materials like corn, sugarcane, and wheat, offers a sustainable alternative to fossil fuels with a lower carbon footprint. Government regulations now require blending ethanol with petrol at varying proportions, from 15 to 85%. Despite corn and sugarcane being the main sources of bioethanol, reports show the feasibility of producing it from algal biomass, particularly lipid-extracted biomass, which can be converted into simple sugars through saccharification and fermented to produce ethanol [77–79].

Recent studies have investigated microalgae’s potential as a bioethanol feedstock due to their rapid growth, high starch and sugar content, and adaptability to different environments. Microalgae-based bioethanol also reduces greenhouse gas emissions and water usage compared to traditional methods, enhancing economic and environmental sustainability and mitigating competition with food production [80–82]. DMB composed of starch and cellulose can be easily hydrolyzed to yield monosaccharides, which are then fermented to produce ethanol [80, 82, 83]. To ensure sustainability, policies and practices must be implemented to minimize these impacts. Overall, bioethanol holds promise as an alternative to fossil fuels, especially with the potential of microalgae and agricultural waste as feedstocks, though addressing environmental concerns is crucial for its sustainable production and utilization.

## Genetic Engineering of Microalgal Strains

Genetic engineering of microalgal strains entails the manipulation of their genetic makeup to augment desirable traits for diverse applications. To enhance any by-product production at a low cost, various molecular techniques have been developed employing microalgal strains. These processes include augmenting photosynthetic efficiency and improving carbon sequestration by directing carbon fluxes toward high-energy compounds suitable for use as hydrogen sources. Improving photosynthetic efficiency in conventional crops is generally challenging [84, 85]; however, microalgae offer a simpler alternative. This method involves knockouts, replacements, or insertions into the photosynthetic system, proving beneficial for enhancing biomass content [86]. Molecular investigations into *Chlamydomonas reinhardtii*, *Synechocystis*, *Synechococcus*, *Myxococcus*, *Chlorella*, and *Anabaena* have been conducted to understand the molecular aspects of biohydrogen production [87, 88] (Table 3). Gene editing emerges as a viable solution, endowing microalgal strains with industrial capabilities For example, genetically modified microalgae exhibit heightened productivity, environmental tolerance, and resistance to insects [22]. Through targeted genetic modifications, researchers can enhance traits such as lipid productivity, stress tolerance, and nutrient uptake efficiency. Techniques such as CRISPR-Cas9 have revolutionized the precision and efficiency of genetic editing in microalgae, facilitating rapid strain improvement. As research progresses, genetic engineering holds the potential to unlock novel microalgal strains with enhanced capabilities, thereby contributing to sustainable solutions for energy, the environment, and healthcare challenges. However, in light of concerns regarding the stability and safety of producing genetically modified microalgae, it is crucial to assess whether they pose any risks to human welfare and environmental health.

**Table 3:** Genetic engineering technique used for improving microalgal products

Serial No	Microalgae species	Genome size (Mb)	Genetic engineering technique	Targeted product	Validation methods	Expression systems	Performance evaluation	Potential applications	Ref.
1	*Auxenochlorella protothecoides*	22.92	Genome scale and core metabolic model	Biodiesel	Sequencing, phenotypic characterization, lipid analysis	Endogenous promoters or heterologous expression constructs	Enhanced lipid productivity, increased lipid content	Biofuel production	[89]
2	*Chlorella pyrenoidosa*	56.99	Core metabolic model	Enhanced pigment production	Phenotypic assays, pigment quantification	Endogenous promoters or heterologous expression constructs	Improved pigment yield	Food coloring, pharmaceuticals	[90]
3	*Chlorella sorokiniana*	58.53	Response to high-density cultivation and UV radiation; fatty acid profiling	Biofuel	Lipid analysis, growth rate measurements	Endogenous promoters or heterologous expression constructs	Increased lipid content, tolerance to environmental stress	Sustainable biofuel production	[91]
4	*Chlorella variabilis*	46.16	Nitrogen deprivation and long-chain alkenes/Genome scale metabolic model	Converting C_16_ to C_18_ fatty acid	Genetic sequencing, lipid profiling	Endogenous promoters or heterologous expression constructs	Enhanced production of desirable fatty acids	Biofuel feedstock optimization	[92]
5	*Chlorella vulgaris*	39.08	Core metabolic model	Lipid accumulation	Lipid quantification, growth rate analysis	Endogenous promoters or heterologous expression constructs	Increased lipid content	Biofuel production, nutritional supplements	[93]
6	*Monoraphidium neglectum*	69.71	Nuclear genetic transformation	Recommended for biotechnological application	Gene knockout confirmation, growth phenotype assessment	Endogenous promoters or heterologous expression constructs	Enhanced biotechnological potential	Bioremediation, pharmaceuticals	[94]

In conclusion, considering both production costs and scalability, further research on genetic engineering aspects aligned with common international ethics is imperative to advance biohydrogen production in microalgae.

## Utilization of De-oiled Microalgal Biomass in Feed Supplement

De-oiled microalgal biomass is a by-product of microalgal biodiesel production, known for its high protein content and essential nutrients. It is being explored as a feed supplement for livestock, aquaculture, and pet food. A recent study found that it can replace fishmeal as a protein source for chickens, improving their growth and egg production. Similarly, using it in fish feed enhances growth performance and feed efficiency, reducing costs and environmental impacts. Challenges remain, like its high fiber content limiting its use for pigs [78]. Current demand for animal feed supplements relies on maize and soybean meal, but there is little research on using DMB. Its high protein content and essential amino acids make it suitable for livestock and aquaculture. Studies show it can replace up to 7.5% of other feed ingredients for pigs and poultry [95]. Ensuring its safety from heavy metals and solvents is crucial [96]. Studies suggest that supplementing diets with microalgal biomass improves growth performance in pigs [96] and marine shrimp [97]. Ju et al. [98] found that it benefits shrimp pigmentation. Juvenile red drum can tolerate up to 10% replacement of fishmeal and soy protein with DMB [99]. *Nannochloropsis* sp. is also a potential source of essential fatty acid (EPA) [100].

Numerous investigations have been conducted to evaluate the protein content derived from microalgal biomass for potential integration into prawn feed [101]. The intricate composition of microalgal cell walls presents a challenge in the digestion process, thereby complicating the utilization of this biomass as a feed source for prawns. Furthermore, the economic feasibility of such pursuits is impeded by significant production costs. Addressing these challenges is imperative to formulate a cost-effective and efficient solution for prawn feed that maximizes the protein content of microalgal biomass. Mitigating the digestive challenges posed by the robust cell wall and optimizing production processes are crucial steps toward achieving a sustainable and economically viable product. A profound understanding of these intricacies is paramount for the development of innovative strategies and technologies, facilitating the utilization of microalgal biomass as a high-quality protein source in prawn aquaculture [13]. Consequently, the inherent challenges associated with the digestion of the robust cell wall and the associated production costs present formidable obstacles in the pursuit of developing an economically viable product.

Despite this, DMB shows great promise in various applications, offering an alternative protein source, improved growth, and reduced reliance on traditional feed ingredients [102]. However, more research is needed to overcome its high fiber content.

## Utilization of Microalgal Biomass as Fertilizer

Microalgae biomass is a sustainable and eco-friendly resource for biofertilizers that enhances soil fertility and promotes sustainable farming. Green microalgae and cyanobacteria are used as biofertilizers to improve soil and plant parameters [103]. Cyanobacteria have been found to produce metabolites that improve soil fertility and quality [104]. These microalgae directly transform atmospheric CO_2_ into organic algal biomass, making them valuable sources of organic matter [105]. Cyanobacteria can fix atmospheric nitrogen, reducing the need for nitrogen fertilizers in crops [106, 107]. Microalgae like *Spirulina platensis* and *Chlorella vulgaris* enhance soil nutrients and promote crop development [103]. Microalgae biomass improves germination, N uptake, and biomass accumulation in crops [108]. Biofertilizers containing microorganisms enhance soil properties and plant growth [8]. DMP waste is a promising biofertilizer rich in nutrients like potassium (K), N, and P [109]. Microalgae-based biofertilizers reduce nutrient losses, enhance soil health, contribute to carbon sequestration [8], promote rice plant development, improve soil fertility, and efficiently adsorb heavy metals, thus making them reliable options in an eco-friendly manner [110, 111].

Improved crop varieties and efficient nitrogen management techniques can supplement chemical fertilizers [112]. Mixing *C. vulgaris* and *Scenedesmus dimorphus* enhances rice plant (*Oryza sativa*) height [111]. Microalgae biomass enhances crop growth and yield, comparable to synthetic fertilizers [113, 114]. Combining DMB waste and inorganic fertilizer increases tomato (*Solanum lycopersicum* L.) plant growth and yield [115]. Regulations are needed for safe microalgae resource utilization [48].

Additional research is imperative to assess the adaptability of microalgae across diverse soil types, crops, and ecological conditions, thereby facilitating their transition to a commercially viable scale. Commercially available microalgae-based products, such as microalgal biostimulants (MBS) and microbial biofertilizers (MBF), hold promise for enhancing crop yields in agriculture. In this context, MBS do not function as MBF and do not directly provide nutrients to the plants. Instead, they can facilitate nutrient uptake by altering the plant rhizosphere and metabolic activities. Consequently, this improvement in nutrient uptake efficiency enhances tolerance to abiotic stresses, resulting in enhanced crop quality [116]. The chemical characteristics of MBS and MBF are crucial for their effectiveness, with variations depending on microalgal species and production procedures. Labels of MBS and MBF typically provide information on mineral elements, amino acids, and phytohormone quantities. Microalgae biomass is rich in micro- and macronutrients, especially nitrogen (N), phosphorus (P), and potassium (K), making it suitable as an organic slow-release fertilizer. For example, *Arthrospira* sp. contains nitrogen, phosphorus, and potassium concentrations of 6.70%, 2.47%, and 1.14%, respectively. Importantly, *Arthrospira* sp. biomass is lead-free, indicating its safety as a plant growth stimulant. The microalgal species, metal ions (such as Lead (Pb), Nickel (Ni), Cadmium (Cd), and Zinc (Zn)), and growth system conditions (pH) influence metal absorption mechanisms. Accurate chemical analysis is crucial to ensure the safety of MBS and MBF derived from microalgae cultivated in wastewater.

An aqueous cell extract of *Acutodesmus dimorphus* was utilized as a biofertilizer on *Solanum lycopersicum* L. plants. The results indicated an increase in seed germination rate, plant growth, and floral parts [117]. The biostimulant activity of *Arthrospira platensis* was evaluated in papaya plants, revealing that root application of these extracts could increase leaf number and area, stem diameter, and plant height [118]. In another study, *A. platensis* biomass was used as an organic biofertilizer in sweet pepper cultivation, resulting in increased yield [119]. Moreover, *Anabaena vaginicola* and *Nostoc calcicola* positively affected cucumber, squash, and tomato plants by increasing their weight, root length, height, and leaf number [120]. Additionally, *Chlorella sorokiniana* improved wheat plant growth through its total dry biomass and plant length, indicating the potential of biostimulants and biofertilizers in microalgae [121]. Another extract from *Chlorella kessleri* was applied to *Vicia faba* plants, showing a significant effect on improved germination, seedling growth, leaf surface area, pigment concentration, and sodium and potassium accumulation in roots and shoots [122]. Furthermore, extracts from *Scenedesmus quadricauda* and *Chlorella vulgaris* exhibited effective biostimulant activity by influencing root traits and genes in *Beta vulgaris*. This activity can contribute to increased nutrient acquisition in the plant [123]. Overall, microalgal biomass can be utilized to improve seed germination, flowering, and fruit yield in various plants through application to the leaf or root system. However, it is crucial to standardize the application type and optimum concentration of algal extracts to achieve a significant effect on plants. Interestingly, microalgae extracts contain various plant growth-promoting compounds, as evidenced by their morphologically based results in crops. Nonetheless, it is recommended to conduct physiologically based experiments to understand the specific chemical constituents involved in crop growth and development.

Numerous studies have highlighted microalgae’s plant growth-promoting compounds, including auxins, cytokinins, betaines, amino acids, vitamins, and polyamines. Concentrations of auxins and cytokinins were analyzed in 24 microalgae strains with cis-zeatin as the most common cytokinin [124]. Microalgae also contain significant concentrations of brassinosteroids and gibberellins. Protein hydrolysates, active components of plant biostimulants, enhance biological activity in crop growth and development [125]. Amino acids present in microalgae are well-known biostimulants that benefit plant growth and agricultural productivity, as well as mitigating abiotic damage. Polysaccharides like glucan found in microalgae promote plant growth by interacting with leucine-rich repeat membrane receptors, activating pathways regulating genes involved in cell proliferation [104].

## Biosorption of Heavy Metals and Dyes by Using De-oiled Microalgal Biomass

Microalgae are not typically recommended for consumption as they tend to absorb harmful metals. However, their exceptional ability to remove metals from water and other contaminants makes them valuable for bioremediation purposes. *Scenedesmus* is commonly used for heavy metal removal in bioremediation studies. *Chlorella* sp. is also capable of removing various heavy metals such as copper, Zn, Pb, mercury, arsenic, Cr, Ni, and Cd. Microalgae species like *Chlorella minutissima* and *Phaeodactylum tricornutum* show high tolerances to Cd and can eliminate Cr (VI). *Chlorella* strain is selected for its ability to thrive in the presence of 11.24 mg Cd/L and remove 65% of the metal when exposed to 5.62 mg Cd/L. Similarly, *Scenedesmus* and *Chlorella* strains demonstrated clearance rates of 48% and 31%, respectively, for 20 mg Cr/L [110, 126].

Dye industrial sewage is a major source of water pollution that reduces sunlight penetration in natural water bodies. This inhibits photosynthesis and increases the demand for oxygen by aquatic life [16]. Adsorption has become the preferred method for removing dyes from wastewater due to its simplicity and scalability. DMB is effective in biosorbing dyes and heavy metals from wastewater [17].

Acid-treated defatted biomass of *S. dimorphus* has the highest adsorption capacity for methylene blue [127]. Others used DMB of *Microspora* sp. for the biosorption of methylene blue, achieving 86% removal within 24 h [16]. Factors such as pH, temperature, contact time, and initial concentration influenced the effectiveness of biosorption. *S. platensis* DMB can absorb more Cr (VI) than its fresh biomass [128]. Chemically modified microalgal waste can selectively biosorb palladium (Pd II) and platinum (Pt IV) [129]. DMB of *Nannochloris oculata* has high adsorption capacities for Cr (III/IV) [130].

## Holistic Biorefinery Circular Bioeconomy Approach

**Fig. 2:**
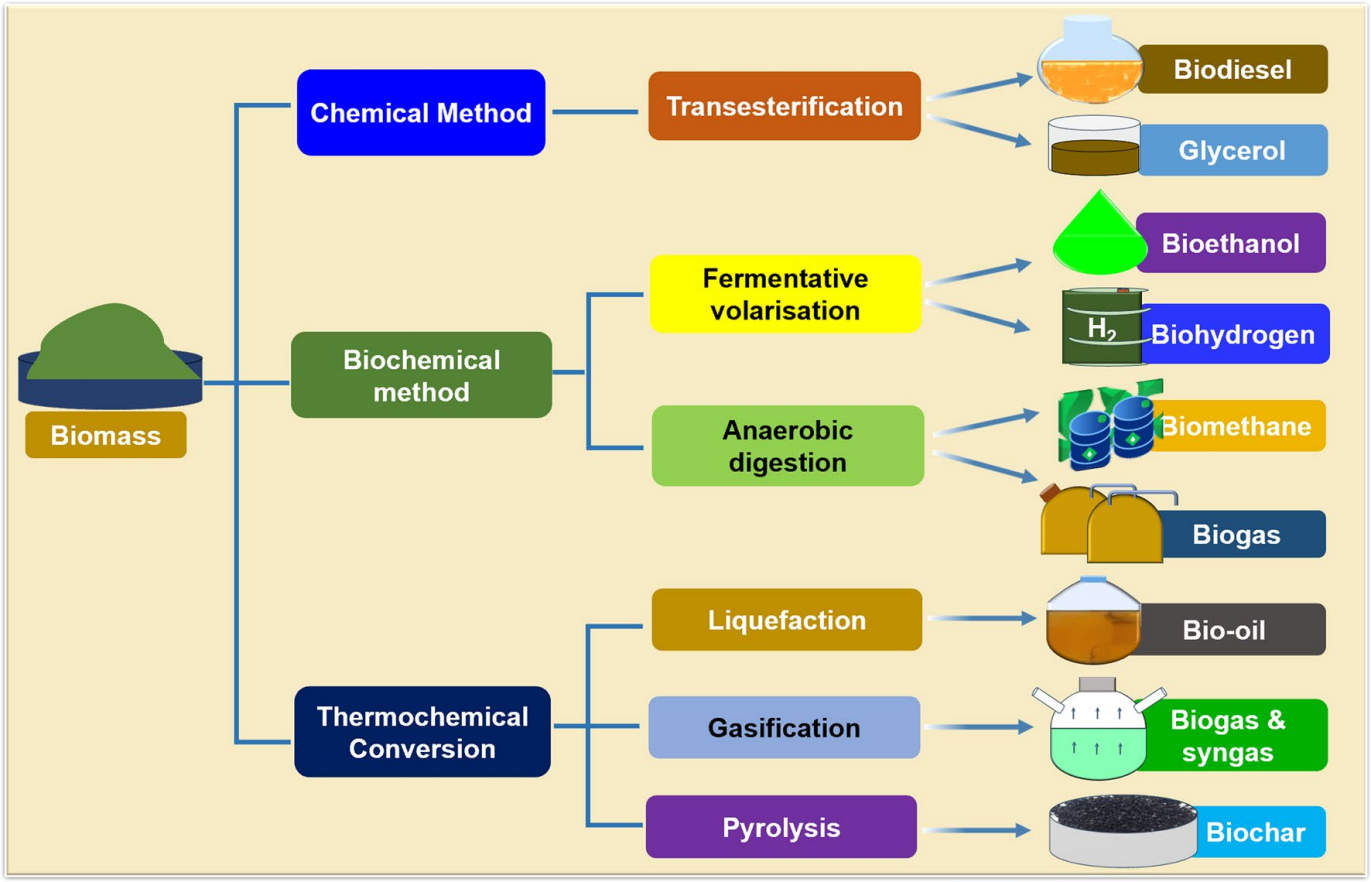
Development of a microalgae biorefinery system for sustainable production of biofuels and other bioproducts. This image represents the process of the circular economy of algal production and the development of microalgae biorefinery systems for sustainable biproduct production, such as biofuel, feed supplements, and fertilizer

Microalgae have gained attention for their potential in biorefineries due to their ability to use nutrients from wastewater. However, current large-scale processes relying on single or dual product systems are not economically feasible. Researchers are working on optimizing material and energy balances to produce multiple products from one microalgae strain. Figure 2 illustrates the microalgae biorefinery system, emphasizing the importance of a circular bioeconomy. This review focuses on circular bioeconomy using wastewater for microalgae cultivation and the production of various products from raw and de-oiled microalgae residues.

Raw algae biomass can produce high-volume, low-quality goods like lipids and biodiesel. To improve efficiency, it is important to produce low volumes of high-value products such as vitamins, proteins, DHA, and carbohydrates. Another income avenue is valorizing de-oiled algae residues into bio-based products [131].

Integration of processes in microalgae-based biorefineries can enhance biofuel and value-added product recovery [132]. The design framework considers microalgal strain, process flow, side products, and primary output. Integrated valorization of microalgal biomass contributes to the bioeconomy. Biodiesel from microalgae oil can reduce CO_2_ emissions by 78%. Biomethane from residual biomass can power biodiesel facilities. Microalgal biorefineries offer biofuel recovery and other product production [3]. A recent study conducted a comprehensive techno-economic assessment of biofuels and other value-added products derived from microalgal biomass within the context of a biorefinery concept [133]. The cost of producing one tonne of algae was found to vary from $150 to $6000, contingent upon the underlying assumptions and methodologies applied. It is noteworthy that closed systems generally exhibit higher operational expenses in comparison to open systems. Specifically, the oil productivity attained through open ponds and PBR cultivation methods incurs costs ranging from $8.52 to $18.10 per gallon and $9.84 to $20.53 per gallon for biodiesel, respectively [134]. The economic implications become apparent when considering the production of 10,000 tonnes of microalgae containing 30% lipids, with an estimated cost of $2.80 per liter, rendering it more expensive than conventional fuels. However, the prospect of co-cultivating different microalgae species emerges as a potential strategy to enhance lipid content, thereby mitigating production costs [135].

Biorefineries convert biomass into fuels, electricity, and chemicals. Design depends on feedstock, technology, platform substances, and desired products. Valorization supports the bioeconomy. Biohydrogen production can yield valuable by-products. Life cycle assessment (LCA) quantifies the environmental impact of bio-based materials. LCA evaluates potential the environmental effects of products. Environmental system analysis tools examine social, technical, and natural systems [136]. Biofuel production pathways are predominantly regarded as sustainable, characterized by low emissions and reliance on natural resources. However, the utilization of microalgae in biofuel production, as highlighted by Lardon et al. (2009), presents environmental non-competitiveness due to elevated energy consumption during harvesting and oil extraction processes [28]. To address this concern, Russo et al. (2016) emphasize the importance of establishing a sustainable energy source by standardizing waste for recycling and reusing, especially from a life cycle assessment (LCA) perspective [137]. Given the various pathways for converting algal biomass to oil, a comprehensive LCA analysis becomes crucial in mitigating the associated demands and challenges. In the investigation of microalgae cultivation, Orfield et al. [138] propose sugarcane as a feedstock, demonstrating its potential to diminish the global warming impact associated with biodiesel production. However, a separate LCA study, conducted by Jez et al. [139], underscores that the production of oil from microalgae is not competitive when compared to other plant sources and fossil fuels due to heightened electricity consumption. Consequently, there is an immediate imperative to conduct thorough LCAs to discern the optimal selection of materials, energy inputs, and environmental impacts of algal biofuels in surpassing the efficiency of fossil fuels and other biomass alternatives.

## Future Perspectives

Wastewater from various sources, including municipal, agricultural, and industrial sources, has been extensively studied for cultivating microalgae. However, most research has been on a lab scale, with only a few recent pilot studies evaluating, for instance, semi-industrial ponds for treating household sewage and found high removal rates for pollutants [140]. Similarly, a year-long pilot-scale PBR with microalgae-treated urban wastewater gave stable results after 10 months [141]. However, more research is needed to assess practicality and economics in real-world conditions. Objectives in the future will involve screening algae species for different wastewaters, designing systems for large-scale cultivation, improving conditions, and developing efficient technologies for harvesting. Safety methods for using microalgae biomass after purification are also crucial.

Microalgae can capture CO_2_ and nutrients efficiently. Incorporating an algal unit in a sugarcane biorefinery can save costs and reduce CO_2_ emissions by co-producing algal biofuel and biochar for enhanced sustainability [142]. The sustainability of the algal biorefinery process is often questioned due to technological requirements, economics, and environmental issues. Integrating process technology for minimal waste and feasibility remains a major challenge [136, 142].

## Conclusion

Microalgae have great potential for supporting long-term circular bioeconomy systems. They can capture CO_2_ and absorb nutrients from various waste forms like solids, liquids, and gases. These abilities advance circular bioeconomy models. To make microalgal biomass production economically viable, different nutrient sources are used, including food waste, animal manure, coal ash, wastewater, CO_2_, nitric oxide, and sulfur dioxide. Developing biorefinery technologies to reduce production costs is crucial for producing valuable goods and biofuels. Other bioproducts like biofertilizers can also be made from microalgae, maximizing their potential while reducing environmental damage from improper waste disposal. Integrating processes in microalgae biorefineries using circular bioeconomy strategies boosts efficiency, profitability, and resource recovery.

## Data Availability

No datasets were generated or analysed during the current study.
